# The role of acylated ghrelin and unacylated ghrelin in the blood and hypothalamus and their interaction with nonalcoholic fatty liver disease

**DOI:** 10.22038/ijbms.2020.45356.10555

**Published:** 2020-09

**Authors:** Xia Liu, Yaoyao Guo, Zhaozhen Li, Yanling Gong

**Affiliations:** 1 Department of Pharmacy, College of Chemical Engineering, Qingdao University of Science and Technology, Qingdao, China; 2 Shandong Luoxin Pharmaceutical Group Stock Co., Ltd., Preparation Department, Linyi, 276000 China

**Keywords:** Ghrelin, Hypothalamus, Insulin resistance, Non-alcoholic fatty liver- disease, Rat

## Abstract

**Objective(s):**

Ghrelin is a brain-gut peptide involved in substance and energy metabolism. To confirm the hypothesis that ghrelin might be involved in non-alcoholic fatty liver disease (NAFLD), a rat NAFLD model was established and the changes of ghrelin were explored.

**Materials and Methods:**

The rats were divided into control and NAFLD groups. The rats in the NAFLD group were fed a high-fat–high-cholesterol (HFHC) diet for 8 weeks. Total ghrelin (TG), acylated ghrelin (AG), unacylated ghrelin (UAG), and hypothalamic AG and its receptor GHSR-1a expression were detected using ELISA, RIA, RT-PCR, and Western blot, respectively.

**Results:**

Plasma UAG, TG, and the ratio of UAG to AG (UAG/AG) decreased, while protein and mRNA expression of hypothalamic AG and growth hormone secretagogue receptor-1a (GHSR-1a) increased in NAFLD (*P<*0.01). Plasma UAG and UAG/AG were negatively associated with homeostatic model assessment insulin resistance (HOMA-IR), while AG positively correlated with HOMA-IR (R^2^=0.6510, *P=*0.005; R^2^=0.8520, *P=*0.000; R^2^=0.5617, *P=*0.013, respectively). Plasma UAG, TG and UAG/AG negatively correlated with serum LDL-C or hepatic triglycerides (TGs) (R^2^=0.7733, P=0.001; R^2^=0.6930, *P=*0.003; R^2^=0.6042, *P=*0.008; R^2^=0.7046, *P=*0.002; R^2^=0.6722, *P=*0.004; R^2^=0.5124, *P=*0.020, respectively). Hypothalamic AG and GHSR-1a positively correlated with HOMA-IR or hepatic TGs (R^2^=0.5116, *P=*0.020; R^2^=0.5220, *P=*0.018; R^2^=0.6074, *P=*0.008; R^2^=0.5127, *P=*0.020, respectively).

**Conclusion:**

It might be that decreased circulating UAG/AG, rather than UAG or AG alone, were involved in IR and liver lipid accumulation in NAFLD. Acylated ghrelin might induce IR and promote liver lipid accumulation via a central mechanism involved in the hypothalamus.

## Introduction

Non-alcoholic fatty liver disease (NAFLD) is defined as a clinical syndrome in which more than 5% of bullous lipids accumulate in hepatocytes, under conditions other than alcohol abuse and other obvious causes (1). NAFLD has drawn increasing worldwide attention for its high incidence and association with obesity (2). Till now, the “multiple-hit” hypothesis for the pathogenesis of NAFLD has been accepted, which considers complex factors acting together to induce NAFLD (3, 4). Such hits include insulin resistance (IR), hepatic lipid accumulation, adipokines secreted from adipose tissue, gut microbiota, and epigenetic and genetic factors. Insulin resistance increases the cellular uptake of free fatty acids (FFAs) and results in hepatic lipid accumulation and progression to advanced liver disease (5, 6). Hepatic lipid accumulation induces lipotoxicity, which results in mitochondrial dysfunction with oxidative stress and activation of inflammatory responses (7). Therefore, IR is still a crucial factor in the pathogenesis and progression of NAFLD.

Ghrelin is a 28-amino-acid peptide that was isolated from rat gastric tissue (8). It is also the only natural endogenous ligand for the growth hormone secretagogue receptor (GHSR) discovered thus far. There are two forms of GHSR: GHSR-1a and GHSR-1b, and GHSR-1a is the active form. It was first reported in 2003 that plasma ghrelin concentrations decreased in NAFLD patients, a circumstance that was related to insulin resistance (9). Furthermore, hepatic ghrelin overexpression was found in patients with nonalcoholic steatohepatitis (NASH), despite the presence of hypoghrelinemia (2). There are two forms of ghrelin in the circulation: acylated ghrelin (AG) and unacylated ghrelin (UAG). Ghrelin O-acyltransferase (GOAT) is the only enzyme for catalyzing acylation. Previous studies have found that only AG has the ability to activate GHSR-1a and exert its activity. However, recent studies have demonstrated that UAG also exhibits activities through a GHSR independent pathway (10). A study reported an association of high serum ghrelin levels with a low risk of developing NAFLD (11). However, they did not clearly indicate whether TG or AG engaged in NAFLD. Controversially, A group studied 75 morbidly obese patients with NAFLD and found that UAG concentration was twofold higher in patients with NASH than in non-NASH patients (12). Mykhalchyshyn *et al.* recently reported that serum AG levels, which can be used as a diagnostic marker for NAFLD development in patients with type 2 diabetes, increased in NAFLD patients (13). Although controversial, it is certain that ghrelin surely plays a role in the pathogenesis and development of NAFLD. Furthermore, few animal studies on ghrelin and NAFLD have been conducted thus far.

The present research prompts the hypothesis that AG and UAG might play an opposite role in NAFLD. As AG can cross blood-brain barriers and is mainly distributed in the hypothalamus of the central nervous system, how does ghrelin system change in the circulation and hypothalamus in NAFLD? Are these changes related to IR and lipid accumulation? Therefore, it is hypothesized that the imbalance of UAG/AG combined with AG adaptive regulation in the hypothalamus might be involved in the pathogenesis and development of NAFLD. In the present study, a rat NAFLD model was established by feeding a high-fat–high-cholesterol (HFHC) diet and identified by serum lipid, transaminase, and hepatic histology. Insulin resistance, circulating TG, AG and UAG, and AG expression in the hypothalamus were detected, revealing the significance of ghrelin in NAFLD.

## Materials and Methods

***Animals and experimental design***

Twenty male Wistar rats, sourced from (Qingdao Daren Fortune Animal Technology Co. Ltd., Qingdao, China), were acclimatized for 7 days in a standard animal room controlled at 22±2 ^°^C and 55±10% humidity under a 12:12 hr light–dark cycle. Concomitantly, we randomized the rats into two groups equally: a control group and NAFLD group. The rats in the NAFLD group were fed a high-fat–high-cholesterol (HFHC) diet for 8 weeks. All rats in both groups had access to tap water *ad libitum* and were kept in cages individually. The experiments were approved by the Animal Care and Use Committee at the Qingdao University of Science and Technology. All research involving animals was conducted according to the Guide for the Care and Use of Laboratory Animals.

***Sample collection***

At the end of the 8-week HFHC feeding, the rats were weighed and anesthetized with an intraperitoneal injection of sodium pentobarbital (65 mg/kg) after having fasted overnight. Blood was extracted by cardiac puncture, and plasma or serum was prepared by centrifugation at 3500 rpm for 15 min. The rats were then sacrificed by cervical dislocation, and the brains and livers were removed immediately afterward. The hypothalamus of each brain was separated carefully and frozen at -80 ^°^C for real-time polymerase chain reaction (RT-PCR) and Western blot (WB) analysis. The left liver lobe was washed with phosphate buffered saline, fixed in 4% formaldehyde, dehydrated in alcohol, and permeabilized in xylene. Finally, the tissues were embedded in paraffin and cut into 5-μm sections for hematoxylin-eosin (HE) staining. The right liver lobe was washed with PBS and homogenized nine times with absolute ethanol under an ice bath condition. The supernatant was then collected after being centrifuged at 2500 rpm for 10 min, and the liver homogenate was stored at -20 ^°^C for determining hepatic TGs.

***Biochemical analysis***

Serum levels of total cholesterol (TC), triglycerides (TGs), high-density lipoprotein cholesterol (HDL-C), low-density lipoprotein cholesterol (LDL-C), aspartate aminotransferase (AST), alanine aminotransferase (ALT), and hepatic TGs were measured using commercial colorimetric kits (Nanjing Jiancheng Bioengineering Institute, Nanjing, China). Fasting plasma glucose (FPG) levels were determined using a colorimetric kit (Nanjing Jiancheng Bioengineering Institute, Nanjing, China), and fasting plasma insulin (FPI) levels were analyzed using an ELISA kit (Nanjing Jiancheng Bioengineering Institute, Nanjing, China). HOMA-IR was used as a marker for assessing insulin sensitivity and was calculated using the following formula (15): 

All these biochemical procedures were performed in accordance with manufacturer’s instructions.

***Hematoxylin and eosin (HE) staining for liver***

The sections were deparaffinized, washed in PBS, and stained with HE (14, 15). The pathological changes of the livers were observed under a digital light microscope (Olympus, Tokyo, Japan). Inflammation lesions in NAFLD livers were semiquantified using a histological scoring system proposed by Kleiner *et al.* (16). The NAFLD activity score was used to evaluate 3 histological features: steatosis (0-3), lobular inflammation (0-3), and hepatocellular ballooning (0-2). The samples were respectively diagnosed as NASH (>5), borderline (3-5), and diagnosed as “not NASH” (<3), according to the scores. Histological scores were evaluated by two pathological experts blinded to the experimental design protocol.

***Determination of plasma*** ***total ghrelin (TG), acylated ghrelin (AG), and*** ***unacylated ghrelin (UAG)***

Plasma TG and AG levels were determined using commercial RIA kits (Linco Research Inc, St. Charles, MO, USA), and the plasma UAG level was calculated by subtracting AG from TG.

***RT-PCR for hypothalamic acylated ghrelin (AG) and its receptor*** ***growth hormone secretagogue receptor-1a (GHSR-1a)***

According to manufacturer’s instructions, the total RNA of the hypothalamus was extracted using an RNase kit (Qiagen, Valencia, CA, USA). 1 μg of total RNA was subjected to reverse transcription by using a first-strand cDNA synthesis kit (BioRad, Hercules, CA, USA). RT-PCR was completed using a Gene Amp 5700 sequence detection system. SYBR Green I was used as a double-strand DNA specific binding dye and determined through continuous fluorescence. The total amplification volume of 20 μl contained 2×PCR Master Mix, 1 μl cDNA, and 5 μmol/L each specific primer. The specific primer series were as follows: AG, forward 5′-TTGAGCCCAGAGCACCAGAAA-3′ and reverse 5′-AGTTGCAGAGGAGGCAGAAGCT-3′; GHSR-1a, forward 5′-GAGATCGCTCAGATCAGCCAGTAC-3′ and reverse 5′-TAATCCCCAAACTGAGGTTCTGC-3′; and housekeeping gene glyceraldehyde-3-phosphate dehydrogenase (GAPDH), forward 5′-CGGCAAGTTCAACGGCACAG-3′ and 5′-ACTCCACGACATACTCAGCAC-3′. The experimental conditions of RT-PCR were as follows: 95 ^°^C for 3 min, 95 ^°^C for 5 sec, 56 ^°^C for 10 sec, 72 ^°^C for 25 sec (39 cycles); 65 ^°^C for 5 sec, and 95 ^°^C for 50 sec. Using Gene Amp 5700 SDS software to analyze the data, relative quantification was calculated by the difference of the threshold cycle between AG or GHSR-1a and GAPDH (ΔC_t_= C_tAG_-C_tGAPDH_ or ΔCt =C_tGHSR-1a_-C_tGAPDH_) (23).

***Western blot (WB) for hypothalamic acylated ghrelin (AG) and its receptor growth hormone secretagogue receptor-1a (GHSR-1a)***

The hypothalamus sample was lysed to extract the protein, and the protein concentration was determined using a bicinchoninic acid assay (Pierce, Rockford, IL, USA). The 50 μg protein samples were fractionated in Tris–tricine gradient gel electrophoresis, transferred to a nitrocellulose transfer membrane and blocked with 5% skim milk for 1 hr in wash buffer. The membranes were incubated with a special rabbit anti-rat AG antibody (Beijing Bioss Biomart, China,1:1000) or GHSR-1a antibody (Beijing Bioss Biomart, China,1:1000) at 4 °C overnight. All transfer membranes were washed five times in TBST (10 mM Tris-HCl, pH 7.5, 150 mM NaCl, and 0.1% Tween 20) for 10 min and incubated with a horseradish peroxidase-labeled goat antirabbit antibody (Beijing Bioss Biomart, China,1:10000) for 1 hr at room temperature. A chemiluminescent peroxidase substrate (ECL, Amersham Biosciences) was coated on the transfer film and exposed to X-ray film (Kodak X-Omat, Rochester, NY, USA). The film was then scanned, and the optical density of the tape was determined using autoradiometry OptiQuant software (Packard Instruments Company, Meriden, CT, USA) and subsequently normalized using WB parallel to GAPDH (17, 18).

***Statistical analysis***

The data in this paper were expressed as means±SD, and they were analyzed using SPSS 23.0 statistical software. An independent sample t-test was used to compare the means, and linear regression was used to determine the correlation between the variables. A significant difference was recognized at *P*<0.05.

## Results

***Identification of the NAFLD model by blood biochemical parameters and liver pathology***

**Figure 1:**
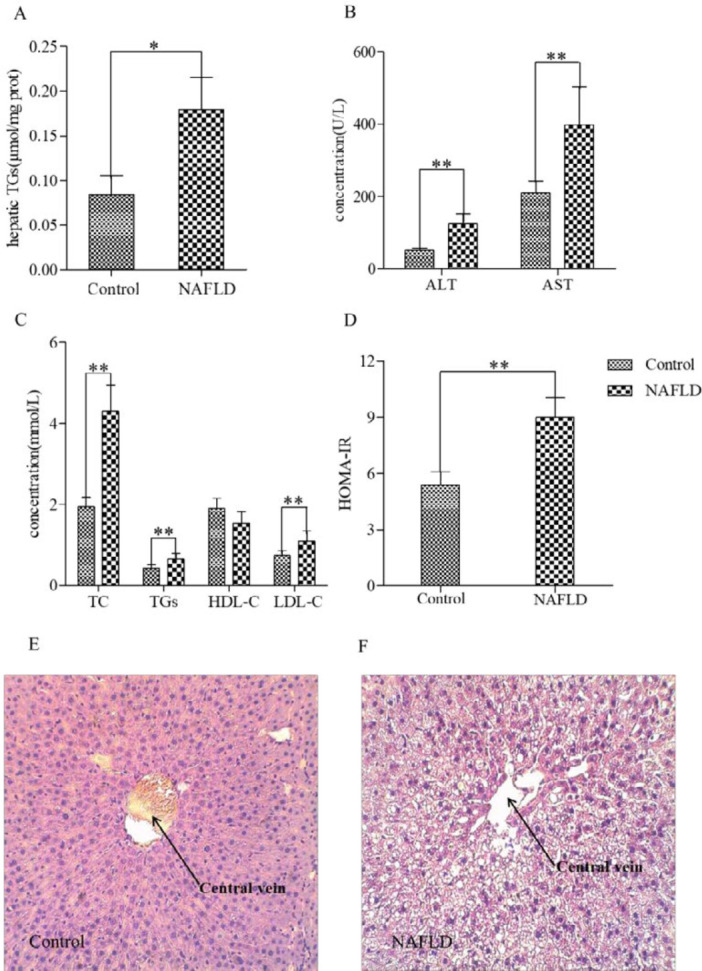
Incidence of NAFLD in rats fed an HFHC diet and associated biochemical changes. The concentrations of hepatic TGs (A), serum ALT, AST (B), TC, TGs, LDL-C (C), and HOMA-IR (D) increased significantly in the NAFLD group. Serum HDL-C (C) had no significant change. Comparison with the control group, *P<0.05, **P<0.01. Liver pathological changes in the control rats (E) and NAFLD rats (F). Typical hepatic steatosis was shown in the NAFLD rats

HFHC: high-fat–high-cholesterol; TGs: triglycerides; ALT: alanine aminotransferase; AST: aspartate aminotransferase; TC: total cholesterol; LDL-C: low-density lipoprotein cholesterol; HOMA-IR: homeostatic model assessment insulin resistance; HDL-C: high-density lipoprotein cholesterol

In the present study, an HFHC diet was fed to rats to establish the NAFLD model and it did produce NAFLD as manifested by its characteristics. To further identify the NAFLD rat model, hepatic TGs, serum AST, ALT, TGs, TC, and liver pathology were observed. Compared with the control group, hepatic TGs (0.1796±0.0357 μmol/mg prot vs 0.0811±0.0158 μmol/mg prot, *P*<0.01), and serum AST (398.20±104.5549 U/l vs 209.80±33.4226 U/L, *P*<0.01), ALT (123.50±28.5161 U/L vs 50.50±7.5019 U/L, *P*<0.01),TGs (4.307±0.6459 mmol/l vs 1.9370±0.2393 mmol/l, *P*<0.01),TC (0.649±0.1384 mmol/l vs 0.428±0.0803 mmol/l, *P*<0.01) in the NAFLD group increased significantly (Figure 1A, 1B, and 1C). Furthermore, the contents of LDL-C increased significantly in the NAFLD group (1.099±0.2424 mmol/l, *P*<0.01) compared with the control group (0.744±0.1149 mmol/l, *P*<0.01, Figure 1C). Otherwise, HDL-C levels had no significant change (1.550±0.2653 mmol/l vs 1.903±0.2540 mmol/l, *P*>0.05, Figure 1C). The stated parameters enabled identifying that HFHC feeding induced hyperlipidemia and liver injury, resulting in a typical change of NAFLD. The HOMA-IR levels increased significantly in the NAFLD group (9.002±1.0603 vs 5.366±0.7368, *P*<0.01, Figure 1D), indicating an insulin resistance induced by HFHC feeding. The HE staining results demonstrated destruction of the hepatic lobule structure with the fatty degeneration of hepatocytes, such as microvesicular and macrovesicular steatosis, and inflammatory cell infiltration in the portal areas (Figure 1F vs 1E), which further confirmed the establishment of the rat NAFLD model.

***Changes of plasma total ghrelin (TG), acylated ghrelin (AG) and unacylated ghrelin (UAG), and their relationships with the biochemical parameters for NAFLD rats***

**Figure 2:**
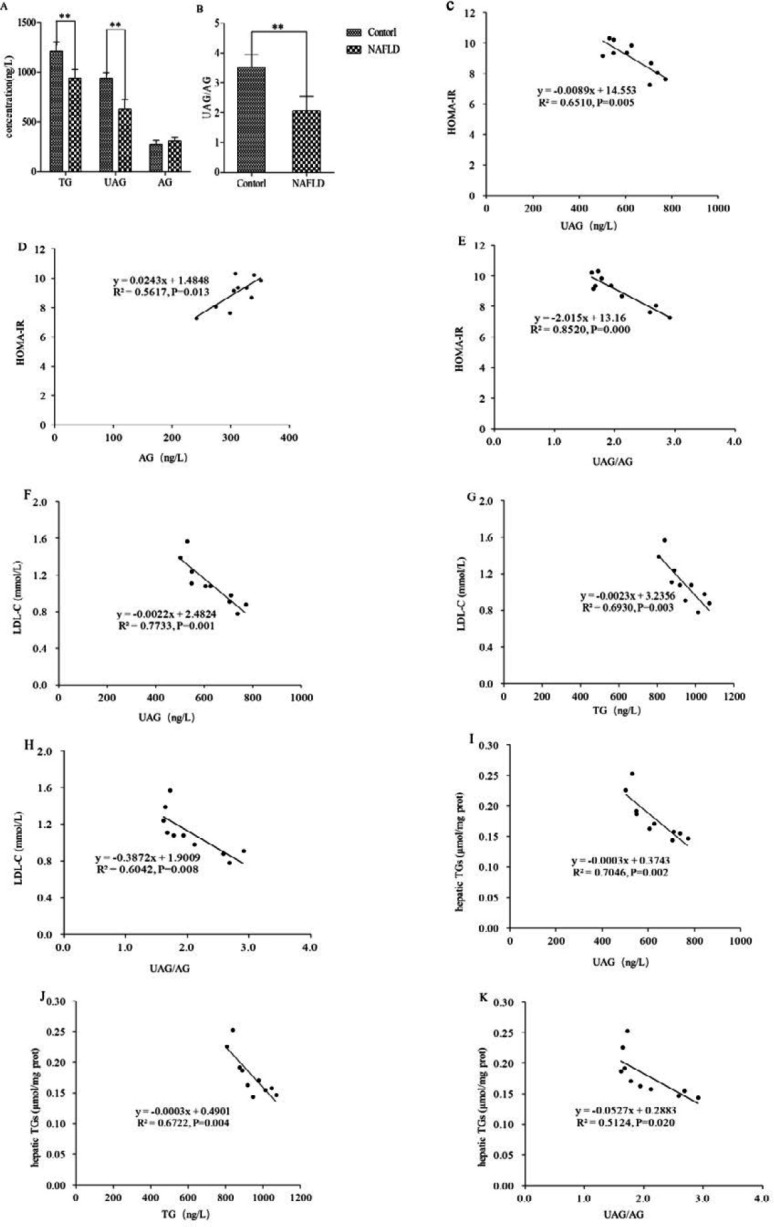
Plasma TG, UAG, AG, and UAG/AG in the NAFLD rats. TG, UAG (A), and UAG/AG (B) decreased significantly while the change of AG (A) had no significance in the NAFLD rats. Comparison with the control group, *P<0.05, **P<0.01. Correlational analysis of plasma ghrelin with serum biochemical parameters and IR. Plasma UAG (C) and UAG/AG (E) showed a negative correlation with HOMA-IR while AG exhibited a positive one (D). Both plasma UAG (F and I), TG (G and J), and UAG/AG (H and K) correlated negatively with serum LDL-C and hepatic TGs, respectively

TG: total ghrelin; AG: acylated ghrelin; UAG: unacylated ghrelin; HOMA-IR: homeostatic model assessment insulin resistance; LDL-C: low-density lipoprotein cholesterol; TGs: triglycerides

Compared with the control group, plasma TG (936.171±88.3639 ng/l vs 1208.496±91.5727 ng/l, *P*<0.01) and UAG (626.855±96.6093 ng/l vs 937.1850±57.5287 ng/l, *P*<0.01) in the NAFLD group were significantly reduced (Figure 2A), while the change of AG (309.314±32.7017 ng/l vs 271.311±44.5514 ng/l, *P*>0.05) had no statistical significance (Figure 2A). Accordingly, the ratio of circulating UAG to AG (UAG/AG) significantly decreased (2.063±0.4857 vs 3.513±0.4348, *P*<0.01, Figure 2B). In addition, the relationships between plasma TG, AG, UAG, and UAG/AG with the biochemical parameters in the NAFLD rats were analyzed here. The results revealed that both plasma UAG and UAG/AG exhibited a negative correlation with HOMA-IR (R^2^=0.6510 and *P*=0.005, Figure 2C; R^2^=0.8520 and *P*=0.000, Figure 2E), while plasma AG showed a positive correlation with HOMA-IR (R^2^=0.5617 and *P*=0.013, Figure 2D). It highlighted that UAG might improve insulin sensitivity while AG induces insulin resistance. Plasma UAG, TG, and UAG/AG showed a negative correlation with serum LDL-C or hepatic TGs, respectively (R^2^=0.7733 and *P*=0.001, Figure 2F; R^2^=0.6930 and *P*=0.003, Figure 2G; R^2^=0.6042 and *P*=0.008, Figure 2H; R^2^=0.7046 and *P*=0.002, Figure 2I; R^2^=0.6722 and *P*=0.004, Figure 2J; R^2^=0.5124 and *P*=0.020, Figure 2K). The above data indicated that it might be the decreased circulating UAG/AG, rather than UAG or AG alone, involved in the IR and liver lipid accumulation in NAFLD.

***Expression of acylated ghrelin (AG) and growth hormone secretagogue receptor-1a (GHSR-1a) in the hypothalamus***

**Figure 3:**
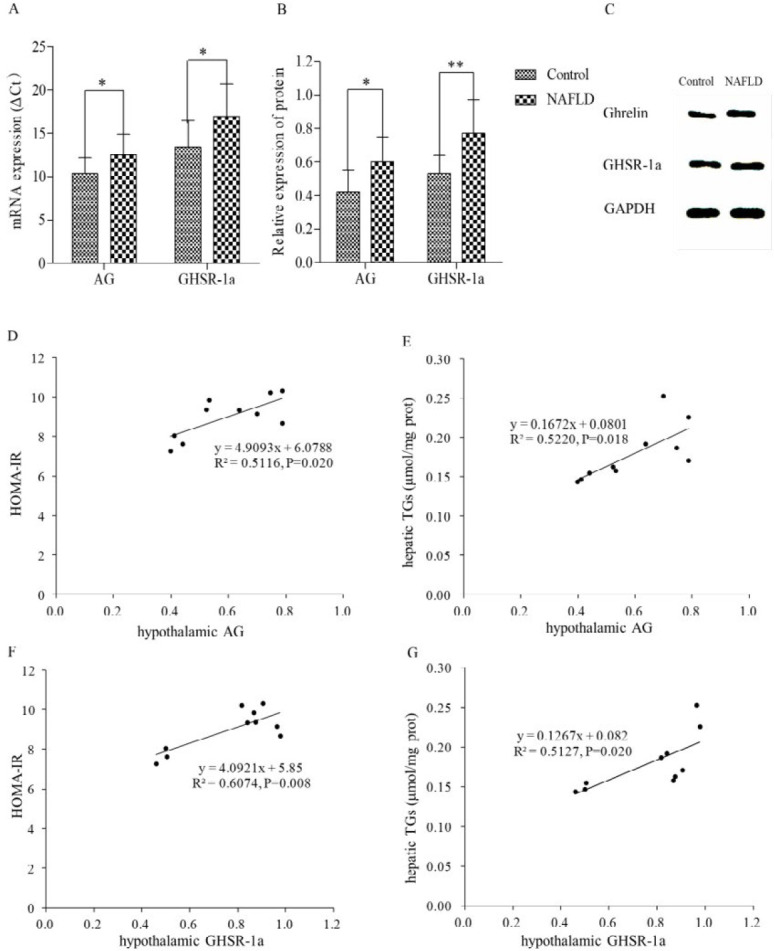
Expression of AG and GHSR-1a in the hypothalamus. The mRNA and protein expressions of AG and GHSR-1a in the hypothalamus increased significantly in NAFLD rats (A, B and C). Comparison with the control group, *P<0.05, **P<0.01. Correlational analysis of hypothalamic AG and GHSR-1a with HOMA-IR and hepatic TGs. Both hypothalamic AG (D and E) and GHSR-1a (F and G) correlated positively with HOMA-IR and hepatic TGs, respectively

AG: acylated ghrelin; GHSR-1a: growth hormone secretagogue receptor-1a; HOMA-IR: homeostatic model assessment insulin resistance; TGs: triglycerides

Compared with the control group, mRNA and protein expression levels of AG and its receptor GHSR-1a in the hypothalamus significantly increased in the NAFLD group (*P*<0.05–0.01, Figures 3A, 3B, 3C). In addition, the relationships between hypothalamic AG and GHSR-1a with HOMA-IR and hepatic TGs in the NAFLD rats were analyzed. The results revealed that both hypothalamic AG and GHSR-1a exhibited a positive correlation with HOMA-IR or hepatic TGs, respectively (R^2^=0.5116 and *P*=0.020, Figure 3D; R^2^=0.5220 and *P*=0.018, Figure 3E; R^2^=0.6074 and *P*=0.008, Figure 3F; R^2^=0.5127 and *P*=0.020, Figure 3G). The results revealed that AG might induce IR and promote liver lipid accumulation via a central mechanism involving the hypothalamus.

## Discussion

In this experiment, feeding rats an HFHC diet for 8 weeks established NAFLD, a disease model that is low cost, stable, safe, and effective. In this experimental model of NAFLD, the serum levels of TC, TGs, AST, ALT, and hepatic TGs in the NAFLD group were significantly higher than those in the control group. In general, the rise of serum transaminase is one of the criteria for diagnosing NAFLD (19). Furthermore, the excessive accumulation of TGs in the hepatocytes was a sign of NAFLD (20). In the state of insulin resistance, insulin inhibits the hydrolysis of TGs, and the excessive release of FFAs from the adipose tissue is absorbed by the hepatocytes and accelerates the synthesis of TGs. In our experiment, the HOMA-IR level in the NAFLD group was significantly higher than that in the control group, indicating that insulin resistance in the NAFLD group led to a decrease in insulin sensitivity. In insulin resistance, oxidative stress occurred in the hepatocytes (21), lipoprotein lipase activity and fat synthesis ability were damaged, and a large number of FFAs were oxidized in mitochondria, causing the FFAs to release from the adipose tissues into the liver, resulting in hepatic fat accumulation and degeneration (22, 23). This is one of the “multiple hits” for the incidence of NAFLD, and the increase of TGs aggravates IR and results in a vicious circle. TGs could be secreted into the blood as LDL-C, and a large number of TGs were synthesized in the liver, inducing increased LDL-C. In NAFLD, the synthesis of phospholipids in the liver decreased, which reduced the synthesis of HDL-C and led to the imbalance of lipids and abnormal blood lipids. Therefore, serum HDL-C decreased, while LDL-C increased in the NAFLD group. The liver pathology showed a typical fatty degeneration in the NAFLD rats. Combined with the biochemical parameters, the NAFLD model was established successfully in our study.

NAFLD is a common chronic liver disease associated with obesity (24). It encompasses a series of liver pathologies, including steatosis, NASH, fibrosis, and cirrhosis, and increases the risk of metabolic diseases (25). Although lifestyle modification or bariatric surgery treats NAFLD effectively (26, 27), no approved drugs specifically for NAFLD are currently available.

Ghrelin, a stomach-derived 28-amino-acid peptide, promotes food intake, weight gain, and adiposity (28, 29). As expected, ghrelin might be associated with lipid accumulation in NAFLD. Previously, it was believed that only AG exhibits bioactivity since it can activate ghrelin receptor GHSR-1a. Nevertheless, the activities of UAG involving gastric motility and lipid and glucose metabolism have since been demonstrated (30-32). Our present study revealed that plasma TG and UAG decreased in the NAFLD rats with little change in plasma AG. As a result, circulating UAG/AG decreased significantly. This indicates that imbalance of UAG/AG, other than UAG or AG alone, might be associated with lipid accumulation in the liver. The data suggest that UAG appears to lower TGs as suggested by a negative correlation between these variables.

Despite no significant increase in circulating AG in the NAFLD rats, the expression of AG and its receptor GHSR-1a in the hypothalamus increased significantly in both mRNA and protein levels. AG acts as an important modulator in central and peripheral lipid metabolism (30). Central administration of AG stimulates the lipogenic program in white adipose tissue and elicits adiposity independently of its food intake effect (33, 34). In addition, central AG promotes hepatic *de novo* lipogenesis in a GH-independent manner (34). Hypothalamic AG and GHSR-1a showed a positive correlation with HOMA-IR and hepatic TGs, respectively. Combined with our results, we postulated that over-expression of AG and GHSR-1a in the hypothalamus might be involved in hepatic lipid deposits and IR, participating in the pathogenesis of NAFLD.

Since IR is one of the severe hits in the pathogenesis of NAFLD, we explored the relationship between IR and circulating ghrelin in our study. Plasma UAG and UAG/AG showed a negative correlation with HOMA-IR, while AG demonstrated a positive correlation, indicating that UAG might improve insulin sensitivity while AG induces insulin resistance. Clinical observation demonstrated that baseline TG and UAG were negatively correlated with HOMA-IR, independent of gender, body mass index, or pharmacological confounders, indicating that UAG prevented IR *in vivo* (35). Another clinical study revealed that AG induces IR, independent of GH, cortisol, or FFAs (36). In high-fat-diet-treated mice, chronic UAG overexpression prevented hyperglycemia and IR and altered insulin signaling (37). Combined with our present findings, it is predicted that the imbalance of circulating UAG/AG, other than UAG or AG alone, might be involved in IR in NAFLD. Therefore, we deduced in the present study that the decrease of circulating UAG/AG promoted hepatic lipid deposit via IR, while AG via a certain role was involved in the hypothalamus. It highlights that restoring the balance of circulating UAG/AG by GOAT inhibitor or blocking the central effect of AG might benefit the intervention of NAFLD.

## Conclusion

The decrease of UAG/AG in the circulation, combined with hypothalamic AG and its receptor GHSR-1a over-expressed, is possibly associated with hepatic lipid deposit and IR in NAFLD. The current findings indicate that targeting the ghrelin system to restore the circulating balance of AG and UAG or block the central effect of hypothalamic AG might be potentially beneficial for the treatment of NAFLD. The detailed mechanism and experimental treatment warrant further exploration.
